# Prognostic roles of hematological indicators for the efficacy and prognosis of immune checkpoint inhibitors in patients with advanced tumors: a retrospective cohort study

**DOI:** 10.1186/s12957-023-03077-8

**Published:** 2023-07-07

**Authors:** Fei Zheng, Qingju Meng, Lei Zhang, Jingli Chen, Liyan Zhao, Zhiguo Zhou, Yibing Liu

**Affiliations:** 1 The Fourth Hospital of Hebei Medical University, Shijiazhuang, China; 2 The First Affiliated Hospital of Xingtai Medical College, Xingtai, China; 3 Hebei Province Pharmaceutical Professional Inspector Corps (Hebei Provincial Vaccine Inspection Center), Shijiazhuang, China; 4 Department of Radiotherapy Oncology, The Fourth Hospital of Hebei Medical University, 12 JianKang Road, Shijiazhuang, 050011 Hebei Province China; 5 Department of Medical Oncology, The Fourth Hospital of Hebei Medical University, 12 JianKang Road, Shijiazhuang, 050011 Hebei Province China

**Keywords:** Advanced tumor, Immune checkpoint inhibitor, Hematological index, Curative effect, Prognosis

## Abstract

**Background:**

Immunocheckpoint inhibitor(ICI) is a major breakthrough in tumor treatment. It can activate the patient’s own immune system and play an anti-tumor role, but not all patients can benefit from it. At present, there is still a lack of effective biomarkers to guide clinical application. The systemic immune inflammation(SII) index reflects the systemic inflammatory state and immune state of patients. Prognostic nutrition index(PNI) can be used to evaluate immune status of patients. Therefore, SII and PNI indexes may have some value in predicting the efficacy and prognosis of immunotherapy, but there is still a lack of relevant research. The purpose of our study was to explore the influence of SII and PNI index on the efficacy and prognosis of immunotherapy.

**Methods:**

A total of 1935 patients treated with ICIs treatment in the Fourth Hospital of Hebei Medical University from November 2016 to October 2021 were retrospectively collected. 435 patients who met the inclusion criteria and did not meet the exclusion criteria. The imaging data, blood results of each patient were collected within 1 week before ICIs treatment. The neutrophil lymphocyte ratio(NLR), platelet lymphocyte ratio(PLR), monocyte lymphocyte ratio(MLR), PNI,systemic inflammatory response index(SIRI),neutrophil-eosinophil ratio(NER) was calculated. The patients were followed up by in-patient, out-patient reexamination and telephone contact, and the efficacy evaluation and survival status were recorded. The deadline of follow-up: January 2021. SPSS-24.0 software was employed for statistical analysis.

**Results:**

Among the 435 patients receiving ICI treatment, 61,236 and 138 patients were evaluated respectively as partial response (PR), stable disease (SD) and progressive disease (PD). The overall response rate(ORR) and disease control rate (DCR) of this cohort were 14.0% and 68.3%, respectively. Median progression-free survival (mPFS) is 4.0 months, The overall survival (mOS) of this cohort is 6.8 months. Multivariate analysis showed that SIRI(Hazard Ratio, HR = 1.304, *P* = 0.014), PNI (HR = 0.771, *P* = 0.019), prealbumin (PAB) (HR = 0.596, *P* = 0.001), and PNI(HR = 0.657, *P* = 0.008) were independent risk factors for PFS and OS, respectively.

**Conclusions:**

Patients with high SIRI value and low PNI value before ICI treatment have shorter PFS. Patients with higher PNI value have better prognosis. Therefore, hematological indicators may become predictors of immunotherapy.

## Introduction

Immune checkpoint inhibitors (ICIs) have been widely used in the clinical treatment of multiple cancer types. However, ICIs cannot benefit all cancer patients, which is still a therapeutic challenge. Study has found that the response rate of ICIs was still lower than 40% in most cancer types [1]. Therefore, it is necessary to screen the most suitable population before immunotherapy. ICI screening methods mainly include immunohistochemistry and genetic testing; however, due to the influence of pathological sampling and the high cost of testing, it is urgent to explore more effective indicators.

SII index is a comprehensive indicator of the number of neutrophils, platelets and lymphocytes in peripheral blood, which can reflect the balance of inflammatory factors and immunity. Previous studies have found that SII index has certain predictive value for the diagnosis of postoperative infectious complications and the long-term prognosis of colorectal cancer patients and for the recurrence of colorectal adenoma [2, 3]. At the same time, previous study has confirmed that the increase of baseline absolute neutrophil count (ANC) is associated with poor PFS and is an independent prognostic factor of PFS in advanced non small-cell lung cancer(NSCLC) with multi-line therapy [4]. PNI is a simple tool calculated by combining the total number of peripheral blood lymphocytes with the concentration of serum albumin (ALB). PNI can be used to evaluate the nutritional level and immune status of patients. Infection is the most common complication after radical resection of gastric cancer, and preoperative low PNI score is a risk factor for postoperative infection [5]. In the retrospective study of advanced NSCLC, ALB ≥ 3.5 g/dl can significantly improve the objective response rate (ORR), PFS and OS of patients treated with ICI, and lower ALB is an independent predictor of poor prognosis of OS [6]. Peripheral blood lymphocytes participate in the destruction and apoptosis of tumor cells and are an important part of anti-tumor immunity. Low lymphocyte count indicates that the body’s anti-tumor immune function is decreased [7].

Although numerous studies have confirmed that single or combination of indicators in peripheral blood are related to ICI efficacy and the prognosis of different malignant tumors, there are few studies on the predictive value of these indicators in advanced solid tumors. Therefore, our study aimed to explore the predictive value of hematological indicators in patients with advanced tumors before immune checkpoint inhibitor (ICI) treatment.

## Methods

### Patients and study design

This was a retrospective cohort study. A total of 1,935 patients diagnosed with advanced tumors from the Fourth Hospital of Hebei Medical University were investigated. The inclusion criteria were as follows: (I) patients aged 18 years or over; (II) those who received pre-treatment blood sampling; (III) patients who underwent at least two cycles of immunotherapy. The exclusion criteria were as follows: (I) patients with fever, systemic inflammation, blood disease, immune disease, cardiovascular or cerebrovascular events, or infection; (II) patients who received blood transfusions within 4 months prior to ICI treatment; (III) patients who underwent local treatment such as radiotherapy and ablation during ICI treatment.

According to these inclusion and exclusion criteria, 453 patients were finally enrolled in the study. Assessment of baseline clinical factors included the patients’ clinical characteristics such as age, gender, pathological type, Eastern Cooperative Oncology Group Performance Score (ECOG-PS), and ICIs were also collected (Table 1). Three patients were admitted to the hospital in a wheelchair, so their height and weight cannot be measured. The study was conducted in accordance with the Declaration of Helsinki (as revised in 2013). The study was approved by Ethics Committee of the Fourth Hospital of Hebei Medical University (No. 2022KY011), but as this study is retrospective, it is not necessary to obtain informed consent from patients.

**Table 1:** Clinical features of patients

Clinical characteristics	Cases	Proportion (%)
Age (years)		
< 62	210	48.3
≥ 62	225	51.7
BMI^a^		
< 23.8	219	50.7
≥ 23.8	213	49.3
PD-L1^b^		
Positive	95	21.8
Negative	63	14.5
Unknown	277	63.7
Sex		
Male	295	67.8
Female	140	32.2
Treatment line		
First line	221	50.8
Second line	103	23.6
Multi-line	111	25.6
Smoking		
Yes	196	45.1
No	239	54.9
Drinking		
Yes	165	37.9
No	270	62.1
Liver metastasis		
Yes	155	35.6
No	280	64.4
Brain metastasis		
Yes	68	15.4
No	367	84.6
The number of organs transferred		
1	255	58.6
2	129	29.7
≥ 3	51	11.7
PS		
0–1	417	95.9
≥ 2	18	4.1
Efficacy evaluation		
PD	138	31.7
SD	236	54.3
PR	61	14
Tumor type		
Lung cancer	167	38.3
Gastric cancer	77	17.7
Esophageal cancer	47	10.8
Colon cancer	33	7.6
Bladder cancer	3	0.7
Bile duct cancer	6	1.4
Gallbladder cancer	4	0.9
Liver cancer	11	2.5
Cervical cancer	15	3.4
Ovarian cancer	3	0.7
Breast cancer	11	2.5
Kidney cancer	7	1.6
Head and neck cancer	13	3
Pancreatic cancer	13	3
Original unknown cancer	13	3
Endometrial cancer	7	1.6
Affiliated organs such as skin	5	1.1
Pathological type		
Adenocarcinoma	217	49.9
Squamous cell carcinoma	105	24.1
Small lung cancer	41	9.4
Malignant melanoma	4	0.9
Sarcoma	8	1.8
Urothelial carcinoma	4	0.9
Neuroendocrine carcinoma	6	1.4
Signet-ring cell carcinoma	3	0.7
Adenosquamous carcinoma	5	1.1
Hepatocellular carcinoma	2	0.5
Cholangiocarcinoma	3	0.7
Large cell carcinoma	2	0.5
Others	30	6.9
Combined treatment		
Yes	373	85.7
No	62	14.3
Drug		
PD-1	416	95.7
PD-L1	19	4.3

*BMI* Body mass index, *PD-L1*, Programmed Cell Death-Ligand 1, *PS* Performance status, *PD* Progressive disease, *SD* Stable disease, *PR* Partial response

Conformed to normal distribution marked as ^a^;PD-L1 ≥ 1%, tumor mutational burden-high (TMB-H), and microsatellite instability-high (MSI-H) /different mismatch repair (dMMR) marked as^b^

### Laboratory testing

Before initial ICI treatment, blood samples were extracted for blood routine and liver function tests. The definition of each ratio is as follows [8]: prognostic nutritional index (PNI) = albumin (ALB) + 5 × lymphoblastic; neutrophil–lymphocyte ratio (NLR) = neutrophil/lymphocyte; systemic inflammatory response index (SIRI) = neutrophil × monocyte/lymphocyte; neutrophil-eosinophil ratio (NER) = neutrophil/eosinophil; platelet-lymphocyte ratio (PLR) = platelet/lymphocyte; monocyte-lymphocyte ratio (MLR) = monocyte/lymphocyte.

### Clinical evaluation and follow-up

Patients received a dynamic computed tomography (CT) scan every two or three cycles of treatment. The response to treatment was evaluated according to the criteria of Response Evaluation Criteria in Solid Tumors (RECIST 1.1), including complete response (CR), partial response (PR), stable disease (SD), and progressive disease (PD). Objective response was defined as CR or PR, while disease control was defined as CR, PR, or SD.

Survival time was defined as the time from the date of receiving ICI to the death of the patient or the last clinical evaluation. The overall response rate (ORR) was defined as the percentage of patients with reduced disease burden to a predefined amount. The disease control rate (DCR) was defined as the percentage of patients who achieved CR, PR, and SD rates. Progression-free survival (PFS) was defined as the time from the treatment of ICI to tumor PD, while overall survival (OS) was defined as the time from the treatment of ICI to death or the last follow-up. The patients were followed up by in-patient, out-patient reexamination and telephone contact, and the efficacy evaluation and survival status were recorded. As of January 1, 2022, all patients had received a post-diagnosis follow-up.

### Observation metrics

The observation metrics were the following: CT results before ICI treatment; blood routine, PAB, ALB, PNI, NLR, SIRI, NER, and Monocyte-lymphocyte ratio (MLR) before ICI treatment; CT results after 2–3 cycles of ICI; PFS and OS after ICI treatment.

### Statistical analysis

Statistical analyses were performed using SPSS-24.0 (SPSS Inc., Chicago, IL, USA). The measurement data of the normal distribution was described as the mean ± standard deviation. The count data were as described as n%, and the inter-group comparison was conducted using the χ^2^ test. Univariate analyses were performed by the *t*-test, χ^2^ test, and log-rank analysis. Multivariate regression analyses were conducted by Cox regression analysis to identify the possible independent prognostic factors. The variables with *p* < 0.05 were screened out in the single factor analysis, and then included in COX regression. HR, we call it risk function value ratio, or risk ratio for short; It is the ratio of the two rates and belongs to one of the relative risk RR values. All *P* values reported are two-tailed. *P* value of < 0.05 was set as the threshold for statistical significance.

## Results

### Clinicopathologic characteristics

A total of 435 patients with tumors following ICI were enrolled in this study. The main tumor types included lung (38.3%), gastric (17.7%), esophageal (10.8%), and colon (7.6%) cancers. The patients’ demographic information and clinical characteristics are shown in Table 1.

### Correlation analysis between peripheral blood indicators and ICI efficacy

Among the 435 patients receiving ICI treatment, 61 were evaluated as PR, 236 were evaluated as SD, and 138 were evaluated as PD. The ORR and DCR of this cohort were 14.0% and 68.3%, respectively. Univariate analysis showed that there were differences between the PR + SD group and PD group in some indicators, including red blood cells (RBC) (*P* = 0.025), hemoglobin (Hb) (*P* = 0.016), absolute eosinophil count (AEC) (*P* = 0.039), ALB (*P* = 0.009), NER (*P* = 0.013), and PNI (*P* = 0.015) after ICI treatment (Table 2). The indicators of RBC (Z =  − 2.681, *P* = 0.007) and Hb (Z =  − 2.863, *P* = 0.004) have significant differences between the PR and SD + PD groups (Table 2). Multivariate analysis showed that the above hematology indicators were negative predictors for ICI efficacy (Table 3).

**Table 2:** Univariate analysis of relationship between peripheral hematology and treatment effect

Hematological index	PD group	PR + SD group	t/Z	*P*	PR group	SD + PD group	*t*//Z	*P*
WBC (× 10^9^/L)	(6.37 ± 3.27)	(6.07 ± 3.02)	− 0.048	0.962	(6.31 ± 3.09)	(5.95 ± 3.04)	− 1.846	0.065
ANC (× 10^9^/L)	(4.44 ± 3.48)	(4.00 ± 2.45)	− 0.350	0.726	(4.50 ± 1.57)	(1.03 ± 2.65)	− 1.744	0.081
ALC (× 10^9^/L)	(1.16 ± 1.11)	(1.34 ± 0.78)	− 1.206	0.228	(1.35 ± 0.80)	(1.25 ± 0.84)	− 0.669	0.503
AMC (× 10^9^/L)	(0.44 ± 0.26)	(0.39 ± 0.24)	− 0.519	0.604	(0.40 ± 0.29)	(0.37 ± 0.23)	− 0.622	0.534
RBC (× 10^9^/L)	(4.91 ± 11.00)	(4.56 ± 7.36)	− 0.025	**0.025**	(4.27 ± 0.55)*	(4.06 ± 0.82)	− 2.681	0.007
Hb (g/L)	(122.30 ± 17.83)	(129.00 ± 26.00)	− 2.401	**0.016**	(134.0 ± 26.8)	(125.95 ± 24.7)	− 2.863	0.004
PLT (× 10^9^/L)	(207.50 ± 122)	(248.61 ± 94)	− 0.601	0.548	(223.0 ± 87)	(229.50 ± 103)	− 0.325	0.746
AEC (× 10^9^/L)	(0.07 ± 0.09)	(0.10 ± 0.12)	-2.068	**0.039**	(0.12 ± 0.16)	(0.09 ± 0.11)	− 1.068	0.286
ABC (× 10^9^/L)	(0.02 ± 0.03)	(0.04 ± 0.02)	− 0.386	0.700	(0.03 ± 0.02)	(0.02 ± 2.65)	− 1.563	0.118
PAB^#^ (mg/L)	(200.67 ± 73.36)^*^	(210.84 ± 69.22)^*^	− 1.398	0.163	(214.47 ± 29.03)*	(205.24 ± 72.30)*	− 0.970	0.334
ALB (g/L)	(40.15 ± 3.36)^*^	(41.10 ± 4.75)	− 2.600	**0.009**	(40.52 ± 3.88)*	(40.75 ± 5.38)	− 0.305	0.761
NLR	(3.49 ± 2.72)	(3.14 ± 2.81)	− 1.437	0.151	(3.30 ± 3.28)	(3.25 ± 2.49)	− 0.458	0.647
PLR	(161.10 ± 124.54)	(183.87 ± 141.75)	− 0.436	0.663	(171.21 ± 133.24)	(175.38 ± 131.65)	− 0.009	0.993
MLR	(0.33 ± 0.23)	(0.30 ± 0.25)	− 1.832	0.067	(0.29 ± 0.33)	(0.30 ± 0.23)	− 0.455	0.649
SIRI	(1.53 ± 1.58)	(1.13 ± 1.73)	− 1.421	0.155	(1.40 ± 1.94)	(1.20 ± 1.50)	− 0.577	0.564
NER	(57.21 ± 96.06)	(40.90 ± 63.54)	− 2.480	**0.013**	(39.92 ± 91.85)	(48.37 ± 68.23)	− 0.319	0.750
PNI	(46.01 ± 5.33)^*^	(47.29 ± 6.05)	− 2.422	**0.015**	(47.75 ± 5.29)*	(47.35 ± 6.20)	− 0.572	0.568

*SD* Stable disease, *PR* Partial response, *Hb* Hemoglobin, *RBC* Red blood cell, *WBC* White blood cell, *ANC* Absolute neutrophil counter, *ALC* Absolute lymphoblastic counter, *AMC* Absolute monocyte counter, *PLT* Platelet, *AEC* Absolute eosinophil count, *ABC* Absolute basophil count, *PAB* Prealbumin, *ALB* Albumin, *NLR* Neutrophil–lymphocyte ratio, *PLR* Platelet-lymphocyte ratio, *MLR* Monocyte-lymphocyte ratio, *SIRI* Systemic inflammatory response index, *NER* Neutrophil-eosinophil ratio, *PNI* Prognostic nutritional index

*P* < 0.05 is statistically significant; conformed to normal distribution marked as *; conformed to homogeneity of variance marked as #

**Table 3:** Multivariate analysis of between peripheral hematology and treatment effect in patients with advanced tumor treated by ICI

SD + PR/PR	B	S.E	Wald	df	Sig	Exp(B)	95% CI for Exp(B)	
							Lower	Upper
SD + PR								
NER	− 0.001	0.001	0.892	1	0.345	0.999	0.997	1.001
PNI	0.051	0.041	1.581	1	0.209	1.053	0.972	1.141
Hb	0.009	0.009	0.934	1	0.334	1.009	0.991	1.027
RBC	0.016	0.064	0.065	1	0.799	1.016	0.897	1.152
PR								
RBC	− 0.024	0.081	0.091	1	0.763	0.976	0.833	1.144
Hb	0.000	0.008	0.002	1	0.965	1.000	0.985	1.016

*CI* Confidence interval, *SD* Stable disease, *PR* Partial response, *NER* Neutrophil-eosinophil ratio, *PNR* Prognostic nutritional index, *Hb* Hemoglobin, *RBC* Red blood cell, *PD-L1* Programmed cell death-ligand 1, *TMB-H* Tumor mutational burden-high, *MSI-H* Microsatellite instability-high, *dMMR* Different mismatch repair

*P* < 0.05 is statistically significant; PD-L1 ≥ 1%

### Correlation analysis between peripheral blood indicators and PFS after ICI treatment

**Fig. 1:**
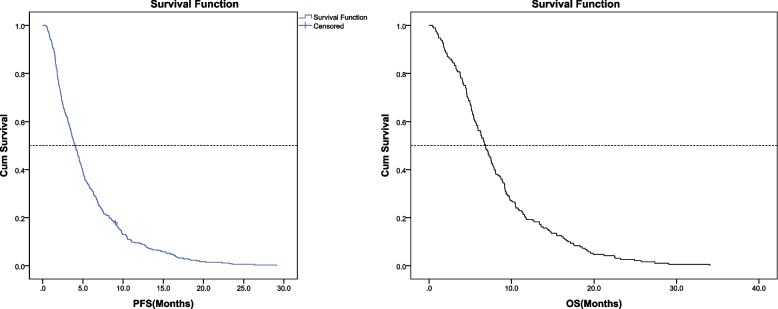
PFS and OS. PFS, progression-free survival; OS, overall survival

**Fig. 2:**
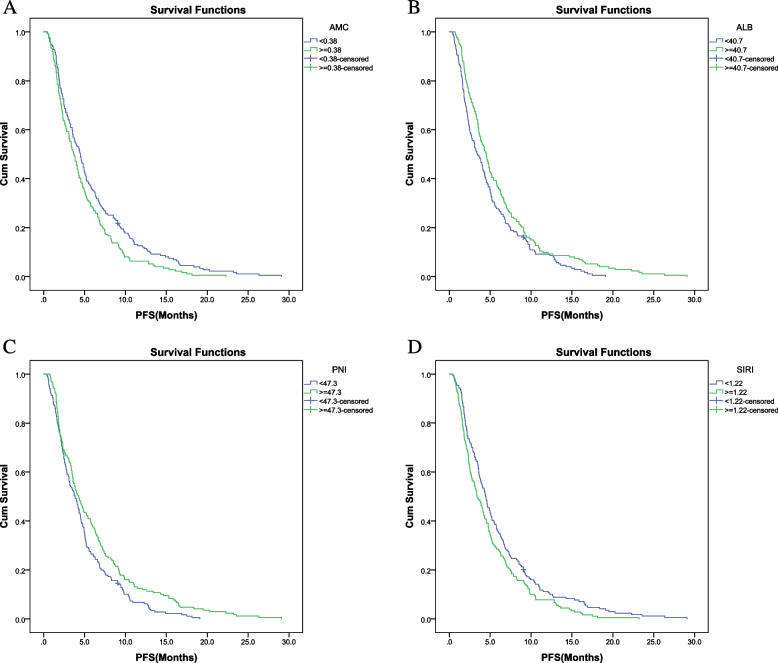
Kaplan–Meier curve of PFS of patients with different AMC, ALB, PNI, and SIRI. PFS, progression-free survival; AMC, absolute monocyte count; ALB, albumin; PNI, prognostic nutritional index; SIRI, systemic inflammatory response index

Until January 1, 2022, 82 patients were evaluated as SD/PR, and 353 patients were evaluated as PD, among which 193 patients died, with median progression-free survival (mPFS) of 4.0 months (95% confidence interval [CI]: 3.5–4.5 months) (Fig. 1). Based on the univariate analysis, blood indicators such as AMC (*P* = 0.009), ALB (*P* = 0.012), PNI (*P* = 0.008), and SIRI (*P* = 0.014) were related to PFS after ICI treatment. Patients with baseline ALB ≥ 40.7 g/L, PNI ≥ 47.3, AMC < 0.38, and SIRI < 1.22 had longer PFS than other patients (Table 4, Fig. 2). Multivariate analysis showed that SIRI (hazard ratio [HR] = 1.304, *P* = 0.014) and PNI (HR = 0.77, *P* = 0.019) were independent risk factors for the PFS of patients. Therefore, patients with a baseline SIRI ≥ 1.611 had a 1.304-times higher risk of disease progression than those with a baseline SIRI ≤ 1.055, and patients with a baseline PNI ≥ 0.959 had a 0.771-times lower risk of disease progression than those with a baseline PNI ≤ 0.620 (Table 5).

**Table 4:** Univariate analysis of hematological indexes and PFS, OS

Inspection index	mPFS (95% CI) months	χ^2^	*P*	mOS (95% CI), months	χ^2^	*P*
WBC (× 10^9^/L)						
< 6.08	4.1 (3.5–4.7)	1.385	0.239	6.9 (6.1–7.7)	0.19	0.889
≥ 6.08	3.9 (3.0–4.7)			6.6 (5.0–8.2)		
ANC (× 10^9^/L)						
< 4.16	4.3 (3.7–4.8)	1.218	0.270	6.9 (6.0–7.7)	0.003	0.956
≥ 4.16	3.4 (2.6–4.2)			6.6 (5.0–8.2)		
ALC (× 10^9^/L)						
< 1.27	3.7 (3.0–4.4)	3.178	0.075	6.4 (5.2–7.6)	1.761	0.184
≥ 1.27	4.3 (3.6–5.1)			7.2 (6.2–8.1)		
AMC (× 10^9^/L)						
< 0.38	4.5 (3.8–5.2)	6.875	**0.009**	7.1 (6.2–8.1)	2.17	0.141
≥ 0.38	3.7 (3.1–4.3)			6.4 (5.1–7.6)		
RBC (× 10^9^/L)						
< 4.09	3.7 (3.0–4.4)	1.178	0.278	6.9 (5.4–8.4)	0.003	0.959
≥ 4.09	4.5 (3.7–5.3)			6.8 (6.0–7.7)		
Hb (g/L)						
< 126	3.6 (3.0–4.2)	1.892	0.169	6.3 (5.2–7.4)	1.802	0.179
≥ 126	4.6 (3.9–5.3)			7.5 (6.5–8.5)		
PLT (× 10^9^/L)						
< 227	4.1 (3.4–4.8)	0.037	0.848	7.0 (6.3–7.8)	1.108	0.292
≥ 227	4.0 (3.4–4.6)			6.4 (4.9–7.8)		
AEC (× 10^9^/L)						
< 0.09	3.7 (3.1–4.4)	0.129	0.720	6.6 (5.3–7.9)	0.3	0.584
≥ 0.09	4.3 (3.7–5.0)			7.2 (6.4–8.0)		
ABC (× 10^9^/L)						
< 0.02	4.3 (3.4–5.2)	0.013	0.909	5.9 (3.6–8.3)	0.448	0.503
≥ 0.02	4.0 (3.5–4.5)			6.8 (6.0–7.7)		
PAB (mg/L)						
< 206.56	3.7 (3.2–4.3)	2.923	0.087	5.7 (4.7–6.7)	16.734	< 0.001
≥ 206.56	4.4 (3.6–5.2)			8.1 (6.7–9.5)		
ALB (g/L)						
< 40.7	3.4 (2.6–4.2)	6.382	**0.012**	5.7 (5.0–6.5)	15.642	< 0.001
≥ 40.7	4.5 (3.9–5.2)			8.7 (7.6–9.8)		
NLR						
< 3.26	4.4 (3.6–5.2)	0.923	0.337	7.4 (6.6–8.3)	3.44	0.064
≥ 3.26	3.4 (2.7–4.1)			5.9 (4.9–7.0)		
PLR						
< 175.23	4.0 (3.3–4.8)	1.336	0.248	6.9 (5.8–8.0)	1.468	0.226
≥ 175.23	4.0 (3.4–4.6)			6.6 (5.3–8.0)		
MLR						
< 0.30	4.5 (3.7–5.3)	3.242	0.072	7.5 (6.6–8.3)	1.789	0.181
≥ 0.30	3.5 (2.9–4.1)			5.9 (4.8–7.0)		
PNI						
< 47.3	3.9 (3.1–4.6)	7.095	**0.008**	5.9 (5.0–6.8)	9.591	0.002
≥ 47.3	4.3 (3.5–5.1)			8.1 (6.2–10.0)		
SIRI						
< 1.22	4.4 (3.8–5.1)	6.102	**0.014**	7.2 (6.5–7.8)	2.379	0.123
≥ 1.22	3.4 (2.7–4.1)			6.4 (5.2–7.6)		
NER						
< 47.72	4.5 (3.8–5.3)	1.346	0.246	7.3 (6.5–8.1)	0.766	0.382
≥ 47.72	3.4 (2.7–4.1)			6.4 (5.0–7.7)	0.19	0.889

*PFS* Progression-free survival, *OS* Overall survival, *mPFS* Median progression-free survival, *mOS* Median overall survival, *WBC* White blood cell, *ANC* Absolute neutrophil counter, *ALC* Absolute lymphoblastic counter, *AMC* Absolute monocyte counter, *Hb* Hemoglobin, *RBC* Red blood cell, *PLT* Platelet, *AEC* Absolute eosinophil count, *ABC* Absolute basophil count, *PAB* Prealbumin, *ALB* Albumin, *NLR* Neutrophil–lymphocyte ratio, *PLR* Platelet-lymphocyte ratio, *MLR* Monocyte-lymphocyte ratio, *PNI* Prognostic nutritional index, *SIRI* Systemic inflammatory response index, *NER* Neutrophil-eosinophil ratio

*P* < 0.05 is statistically significant

**Table 5:** Multivariate analysis of PFS and OS in patients with advanced tumors treated with ICI

PFS/OS	B	SE	Wald	df	Sig	Exp(B)	95% CI for Exp(B)	
							Lower	Upper
PFS								
SIRI	0.265	0.108	6.029	1	**0.014**	1.304	1.055	1.611
PNI	− 0.260	0.111	5.462	1	**0.019**	0.771	0.620	0.959
OS								
PAB	− 0.517	0.158	10.709	1	**0.001**	0.596	0.437	0.813
PNI	− 0.419	0.157	7.119	1	**0.008**	0.657	0.483	0.895

*PFS* Progression-free survival, *OS* Overall survival, *CI* Confidence interval, *SIRI* Systemic inflammatory response index, *PNI* Prognostic nutritional index, *PAB* Prealbumin, *ALB* Albumin

*P* < 0.05 is statistically significant

### Correlation analysis between peripheral blood indicators and OS after ICI treatment

**Fig. 3:**
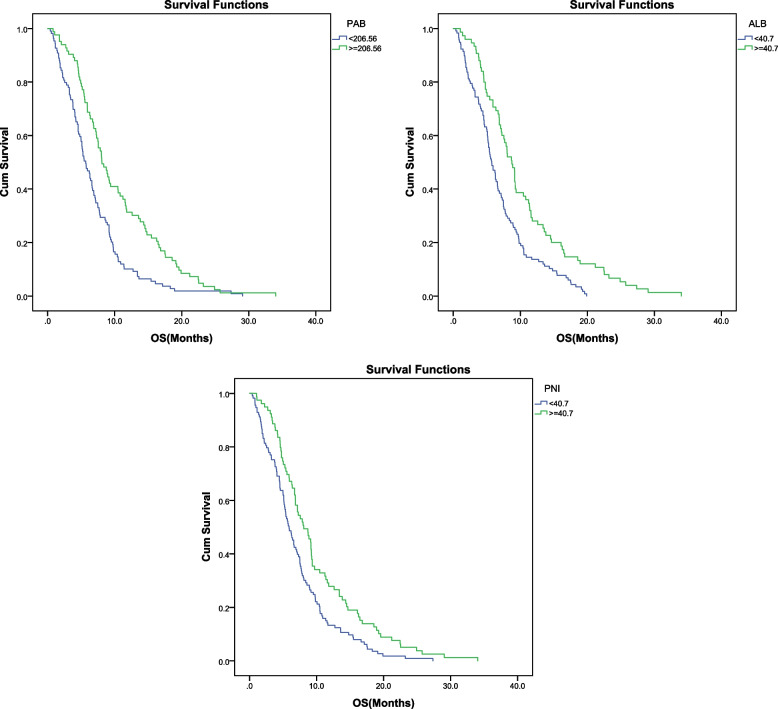
Kaplan–Meier curve of OS of patients with different PAB, ALB, PNI. PFS, progression-free survival; OS, overall survival; PAB, Prealbumin; ALB, Albumin; PNI, prognostic nutritional index

The mOS of this cohort is 6.8 months (95% CI: 6.0–7.6 months). Univariate analysis showed that peripheral blood indicators such as PAB (*P* < 0.001), ALB (*P* < 0.001), and PNI (*P* = 0.002) were associated with the OS of advanced tumor patients receiving ICI treatment (Fig. 3). Multivariate analysis showed that the baseline PAB (HR = 0.596, *P* = 0.001) and PNI (HR = 0.657, *P* = 0.008) were independent risk factors for predicting the OS of patients. Patients with baseline PAB ≥ 0.813 and PNI ≥ 0.895 had a 0.596 and 0.657 times lower risk of death than those with baseline PAB ≤ 0.437 and PNI ≤ 0.483, respectively (Table 5).

### Correlation between NLR and ICI treatment prognosis at different cut-off values

We also explored the relationship between the NLR and prognosis of patients receiving ICI treatment under the median NLR critical values of 3, 3.26, and 5, respectively. Moreover, no correlations were observed between the NLR and PFS/OS of patients receiving ICI treatment (*P* > 0.05) (Table 6).

**Table 6:** Correlation between NLR and prognosis at different critical values

OS/PFS	Critical values	B	SE	Wald	Sig	Exp(B)	95% CI for Exp(B)	
							Lower	Upper
OS	NLR = 5	0.104	0.122	0.721	0.396	1.109	0.873	1.410
	NLR = 3	0.140	0.109	1.663	0.197	1.150	0.930	1.423
PFS	NLR = 5	0.248	0.161	2.370	0.124	1.282	0.934	1.758
	NLR = 3	0.154	0.156	0.984	0.321	1.167	0.860	1.584

*PFS* Progression-free survival, *OS* Overall survival, *NLR* Neutrophil–lymphocyte ratio, *SE* Standard error

*P* < 0.05 is statistically significant

## Discussion

Study has shown that AEC not only participates in hypersensitivity reactions and immunity to parasites but also plays an important role in anti-tumor [3]. In a mouse model and colorectal cancer specimens’ study, Reichman et al. found eosinophils were recruited to the tumor microenvironment (TME), which supported prolonged eosinophil survival and cytotoxic T lymphocytes (CD8 + T-cell)–independent antitumorigenic activities [9]. Previous studies have demonstrated that NER is a negative predictor of anti-tumor response in ICI-treated advanced lung cancer, melanoma, and renal cancer [10–12]. However, NER (cutoff value = 24.32, *P* = 0.051) was strongly correlated with the response to anti-programmed cell death protein 1 (PD-1) treatment in most patients with advanced tumors; however, there was no statistical difference [13]. Our study found a correlation between the baseline AEC (*P* = 0.039) and NER (*P* = 0.013) and the efficacy of ICI treatment, suggesting that it may be easy to control the tumor after ICI treatment in patients with high baseline AEC values and low NER values, without affecting the efficacy.

Lymphocytes play a central role in the tumor immune system. Tumor-associated antigens are presented by dendritic cells (DCs) and then activate T lymphocytes and enter the tumor Immune microenvironment (TME) to kill tumor cells [14]. Our study found that the level of baseline AMC and SIRI were related to the PFS of patients with advanced tumors; the higher the baseline SIRI level, the shorter the PFS of the patients. A pan-cancer study of 107 patients treated with single-agent ICIs showed that a higher baseline AMC level was significantly associated with a shorter PFS [15]. Similar conclusions were also obtained in this study, which supports that the level of baseline AMC can be used as a predictor of ICI treatment. In advanced urothelial carcinoma patients treated with pembrolizumab, the pretreatment SIRI level was associated with OS [16]. The level of baseline SIRI was also found to be associated with PFS and OS in advanced renal cancer patients who received at least second-line nivolumab [17]. However, the level of baseline SIRI was not found to be associated with prognosis in extensive-stage small cell lung cancer (SCLC) patients with first-line chemotherapy combined with ICIs [18]. Therefore, a large number of clinical studies are still needed to verify the predictive value of SIRI for the prognosis of advanced tumor patients treated with ICIs.

Hypoxia can affect the gene expression, cell metabolism, and biological process of tumors. Previous study has found that tumor hypoxia is one of the main causes of drug resistance [19]. Our study found that the levels of baseline Hb and RBC before ICI treatment were related to the curative effect of patients. A high level of baseline Hb and RBC may indicate a better efficacy of ICI treatment. In a meta-analysis of lung cancer involving 22,719 patients, it was found that the lower the Hb level, the shorter the OS of NSCLC or SCLC patients, indicating that reduced Hb levels were significantly associated with shorter OS in lung cancer patients [20]. Hb level before ICI treatment is an independent prognostic biomarker for PFS and OS in patients with advanced gastric cancer, and correcting anemia before ICI treatment can improve the survival rate of gastric cancer patients [21].

ALB is synthesized by the liver and is related to the nutritional status and inflammatory status of the body [22]. Poor nutritional status will affect the body’s immune system. Like ALB, PAB is a negative acute-phase protein synthesized by the liver, and low levels of PAB impair the immune system, inhibit cell-mediated immune function, and lead to increased metastasis [23]. Our study found that patients with high levels of baseline ALB and PNI had a longer PFS and OS than those with low levels of baseline ALB and PNI. PNI was also an independent prognostic factor for PFS (HR = 0.784, *P* = 0.019) and OS (HR = 0.657, *P* = 0.008). Our study findings were consistent with previously reported findings [24–29]. Our study also found that the PAB level (HR = 0.596, *P* = 0.001) was an independent factor affecting the OS of patients with ICI treatment. To our knowledge, this study is the first to find that the level of baseline PAB is associated with prognosis of patients after ICI treatment. Li et al. reported that PAB is an independent factor of OS in NSCLC patients receiving immunotherapy; however, they mainly explored the PAB level after the second cycle of immunotherapy [30]. There are still no reports on the level of baseline PAB.

Since neutrophils are the main mediators in the inflammatory process, NLRs play a major role in tumor mutational burden (TMB) immunosuppression [31–33]. Lymphocytes play a central role in the tumor immune cycle and can kill tumors in the TME [8]. Several studies have also shown that NLR is associated with the prognosis of patients receiving ICI treatment. A meta-analysis of pan-cancer species found that the PFS (HR = 1.81, 95% CI: 1.36–2.41) and OS (HR = 2.26, 95% CI: 1.68–3.03) [34] of patients with high baseline NLR levels were significantly shorter than those with low baseline NLR levels. Furthermore, in a retrospective cohort study of 1,714 patients with 16 cancer types treated receiving ICI, high baseline NLR levels were significantly associated with shorter OS and PFS, low response rates, and low clinical benefit rates [35]. In our study, we analyzed NLRs with different cutoff values (including 3.26, 3, and 5) and found that the NLR had no predictive value for the prognosis of advanced tumor patients treated with ICI. The reason may be that the sample size of this study was small, or that the best predictive cutoff value was not found.

The present study had some limitations. There were relatively small numbers of patient observations in some of the subgroup analysis, and the small number of patients, no correction for multiple testing was performed. We will conduct a multicenter prospective study to strictly enforce inclusion and exclusion criteria for included patients in future studies.

## Conclusions

We found SIRI and PNI were independent prognostic factors of PFS in patients treated with ICIs, and PAB and PNI were independent prognostic factors of OS in patients receiving ICI treatment. Patients with high PAB or PNI values had a better prognosis.

## Data Availability

Not applicable.
